# A Novel Two-Week Dynamic HIIT Protocol Improves Roller Skiing Speed and Metabolic Efficiency in Trained Cross-Country Skiers: A Pilot Study

**DOI:** 10.3390/jfmk10040407

**Published:** 2025-10-20

**Authors:** Marcis Jakovics, Edgars Bernans, Raivo Saulgriezis, Inese Pontaga

**Affiliations:** 1 Latvian Academy of Sports Education, Riga Stradins University, Dzirciema 16, LV-1007 Riga, Latvia; marcis.jakovics@rsu.lv (M.J.); rsaulgriezis@edu.riga.lv (R.S.); inese.pontaga@rsu.lv (I.P.); 2 Sports Healthcare Research Center, Riga Stradins University, LV-1006 Riga, Latvia

**Keywords:** dynamic high-intensity interval training, maximal oxygen uptake, cross-country skiing, skiing speed, tapering

## Abstract

**Background**: The aim of the study was to investigate the effects of a novel two-week dynamic high-intensity interval training (HIIT) protocol, characterized by fixed-load and variable-time intervals (“two times up to ten minutes”), on performance and metabolic adaptations in well-trained cross-country skiers. **Methods**: Ten qualified skiers (six males, four females) completed six interval training sessions over two weeks. Pre- and post-intervention tests were performed to assess maximal oxygen consumption (VO_2max_) and ski speed reached, blood lactate concentration, respiratory gas exchange data, and body weight. **Results**: Maximal speed at VO_2max_ increased significantly from 13.5 ± 2.16 to 14.8 ± 1.7 km/h (*p* = 0.0196; Cohen’s d = 1.06). VO_2max_ itself was retained (*p* > 0.05), equivalence testing confirmed stable values within a ±2.8 mL/kg/min margin. Time to reach RER = 1.0 improved significantly across sessions (*p* = 0.021), indicating enhanced metabolic efficiency. Body weight decreased modestly but statistically significantly by 0.54 kg (*p* = 0.016). **Conclusions**: The dynamic HIIT protocol improved maximal performance (speed at VO_2max_ by 32.9%) and metabolic efficiency in trained skiers without altering VO_2max_. These findings support the usefulness of flexible, individualized HIIT models to enhance aerobic endurance, especially for athletes at risk of performance plateaus.

## 1. Introduction

Maximal oxygen consumption plays a significant role in modern sports science and medicine as a functional biomarker for assessing the cardiovascular system’s functions [1,2]. It is a widely described marker to evaluate the risk of cardiovascular events [3]. The same is true in sports science. In cross-country skiers, the result of competitions significantly correlates with peak oxygen consumption as discovered by Sanbakk et al. While analyzing the differences between national and international level skiers it became clear that the efficiency and technique of both groups of athletes were at a similar level; however, a measurable difference was detected in aerobic capacity and peak oxygen consumption, where international-level athletes showed better results (*p* < 0.05) [4]. In investigations where this correlation was not visible, it has been shown that technical aspects such as efficiency compensated for it, as studied in cyclists by Luciano et al. [5] and in runners by Moses et al. [6].

By studying the characteristics of cross-country skiing and the functioning of energy systems when engaged in this sport, Losnegard et al. have investigated a direct connection between the ability of athletes to perform short, intense repetitions, in which excess post-exercise oxygen consumption (EPOC) is observed, the rapid recovery of metabolic and oxygen uptake kinetics after such efforts, and results of competitions in cross-country skiing [7].

In the current literature, researchers have recognized that high-intensity, maximal oxygen consumption interval training is an effective way to improve VO_2__max_. This is confirmed by Bacon et al. in their meta-analysis [8] and emphasized even more by Storoschuk and colleagues in their recent work on Zone 2 training vs. HIIT [9]. The studies of Norwegian author Helgerud J. determined that the 4 × 4 high-intensity interval training method was more effective than moderate-intensity training [10]. Sloth et al. and Gist have also confirmed the effectiveness of sprint interval training on VO_2max_ enhancement in their meta-analyses [11,12]. Gibala and McGee showed that even very short high-intensity interval training (HIIT) protocols, involving only about 15 min of intense work over two weeks, can induce metabolic adaptations comparable to more traditional endurance training [13]. More data on mitochondrial and cardiovascular adaptations with low-volume HIIT supports that high intensity can “replace” some of the volume of longer moderate-intensity work [14]. Seiler over the years has argued that HIIT should not be considered in isolation but in the context of the total training load and intensity balance across the season. The polarized load distribution model is widely described in research and used by practitioners as it shows importance of complementary low-intensity loads in effective HIIT usage [15,16,17]. However, the most recent research still indicates the need to continue studying high-intensity interval training to find the most effective model as not every model has been tested. Within the wide range of HIIT types training outcomes can be highly variable across individuals as Wen described in her meta-analysis [18]. This invites researchers to continue working and improve their understanding of this training process.

According to J. B. Kreher et al., it is known that up to 60% of professional athletes have experienced overreaching at some point in their careers, whereas true overtraining syndrome is far less common, affecting fewer than 5% of athletes. Therefore, the optimization of training load dosing is considered a significant and topical problem in modern sports and physical activity science [19].

There is little question that HIIT is an essential component of a comprehensive training program for aerobic endurance athletes. Nevertheless, the distribution of the specific intensity of training and optimal types of interval training sessions to enhance performance are still unclear [20].

High-intensity interval training interventions of short duration have gained attention for their efficiency and physiological impact, especially in athletic populations. Recent studies have demonstrated that even a brief training cycle—commonly referred to as a “shock cycle”—can elicit significant improvements in endurance performance and cardiovascular fitness. For example, the Salzburg 10/7 HIIT Shock Cycle Study validated a protocol involving 10 HIIT sessions over 7 consecutive days, leading to measurable gains in VO_2_max and time-trial performance among trained individuals [21]. Similarly, previous short-term interventions with as few as six sessions across two weeks have shown notable effects on mitochondrial adaptations, aerobic capacity, and insulin sensitivity in both recreational and trained populations [22,23]. These findings support the use of condensed, high-intensity training blocks as a viable approach for studying acute physiological adaptations and performance responses.

The dynamic HIIT model proposed in this study differs from traditional protocols, where interval durations are fixed, and athletes adjust intensity to meet predetermined time targets. Such fixed-time approaches can lead to inconsistencies in training load—some intervals may be too easy, others excessively demanding—potentially limiting adaptive responses or increasing fatigue risk. In contrast, our dynamic protocol keeps exercise intensity constant while allowing interval duration to be the variable based on individual tolerance, aiming to minimize load mismatches and reduce the risk of non-functional overreaching.

We hypothesized that the novel dynamic HIIT protocol, combining fixed-load with variable-time intervals, would minimize the risk of non-functional overreaching while eliciting significant improvements in aerobic performance characteristics in well-trained skiers.

The study aimed to investigate the effects of a novel two-week dynamic high-intensity interval training (HIIT) protocol, characterized by fixed-load and variable-time intervals (two times up to ten minutes), on performance and metabolic adaptations in well-trained cross-country skiers.

## 2. Materials and Methods

### 2.1. Participants

Four female (age 18 ± 2 years; VO_2max_ 51.2 ± 2.2 mL/kg/min; body mass 63.18 ± 3.58 kg) and six male (age 17 ± 1.67 years; VO_2max_ 58.57 ± 2.81 mL/kg/min; body mass 74.76 ± 3.63 kg) skiers participated in this study. The investigation was conducted by the ethical standards established by the Helsinki Declaration and approved by the LASE Ethical Committee (3/51813). Before any data collection sessions, all participants received an explanation of the study procedures. They had the opportunity to ask questions and sign the study participant’s consent form. For minors, written consent from parents was also obtained.

The inclusion criteria for the study were as follows:

- Consent to participate in the study.
- Age 12–45 years.
- The training volume of the participants was 7 h or more a week for the last year.
- Sufficient skills to perform the predicted load on the treadmill ergometer.
- VO_2max_ for male participants > 50 mL/kg/min, and for females > 40 mL/kg/min.
- Not taking any medication.
- Passed pre-participation medical screening in the last 12 months.

Exclusion criteria:

- Uncontrolled arrhythmia causing symptoms or hemodynamic compromise.
- Syncope.
- Acute respiratory virus infection in the last 2 weeks.
- Uncontrolled asthma.
- Chest pain typical of ischemia.
- Confusion.

### 2.2. Procedures

**Figure 1:**
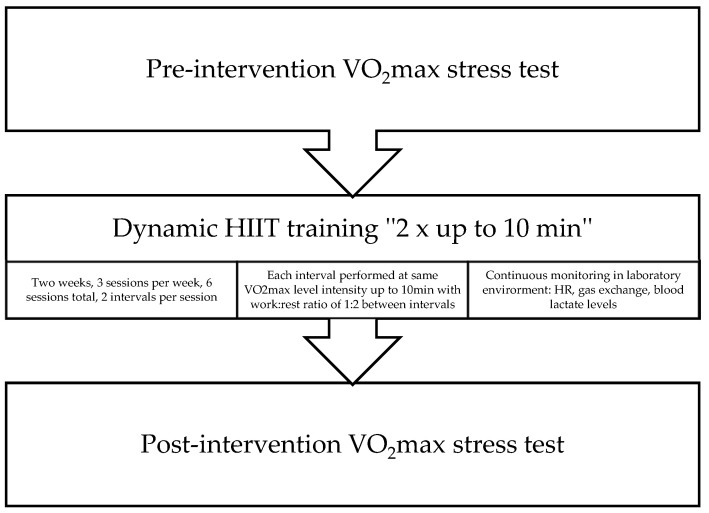
The model for testing and interval training used in the study.

The current study was designed to examine the effect of a novel, dynamic “two times up to ten minutes” HIIT protocol on VO2_max_, body mass, maximal aerobic power, anerobic power, and aerobic capacity. The model for this experiment is represented in Figure 1. Training loads outside of studied intervals were partially regulated, coaches still prescribed the total volume as per the athlete’s plan, but no Zone 4 or higher (more than 80% of HRmax, determined during initial testing) work was allowed during the two-week period. This study used a prospective, longitudinal paired study design.

All exercise tests and interval training sessions were performed in a laboratory setting. Before the start of the experiment, anthropometric data were collected: the body mass of the athletes in underwear was determined in kilograms (kg) using scales (“Tanita” Model: MPB 300K100, Seoul, Republic of Korea), which was later needed to calculate the relative maximum oxygen consumption. Caloric or macronutrient intake or changes in body composition was not measured. Next, the research participants were invited to familiarize themselves with the treadmill ergometer (“Lode” Model: Valiant Ultra 250, Groningen, The Netherlands) and roller skis (“Ski-go” Model: skate Nr 3, Kiruna, Sweden). At the end of every test and interval, immediately post-exercise, blood lactate level was measured using a spectrometer (“EKF diagnostic” Model: Biosen C-line, Barleben, Germany). Breath by breath gas analysis was performed in exercise tests and every interval session to determine respiratory and indirect calorimetry values (“Vyaire” Model: Vyntus CPX, Houten, The Netherlands). Before the start of any tests, a calibration of the gas analyzer was performed according to the manufacturer’s guidelines to ensure the most precise measurements.

#### 2.2.1. Warm-Up

Before exercise tests and interval sessions participants performed a standardized warm-up on a bicycle ergometer (“Lode” Model: Corvial, Groningen, The Netherlands) for 20 min, wearing a heart rate monitor (“Polar” Model: H10, Kempele, Finland) with a heart rate of 120 ± 5 bpm. A 5 min treadmill warm-up was performed to familiarize participants with the conditions.

#### 2.2.2. Exercise Testing

During the warm-up part on the treadmill the test starting load was determined using live respiratory gas exchange data, particularly RER, with an aim to reach 1.0, which corresponds to ventilatory threshold 2 (VT2), a vigorous intensity according to Macintosh and colleagues [24]. This physiological phenomenon is increasingly used in the context of metabolic flexibility and metabolic reaction to different loads [25,26,27]. After determining this intensity, the load was gradually reduced to a complete break, and the participant was informed that the maximal oxygen consumption test would start imminently. This protocol tries to implement recent ideas by Davic C. Poole and Andrew M. Jones, where the idea of performing a high-intensity bout above critical power (CP), which corresponds to VT2, is suggested as optimal to determine VO_2_max [28].

The test was performed at determined incline of 6% and the only requirement for the participant was to cover as much distance as possible within ten minutes. A self-paced maximal effort to volitional fatigue at the intensity eliciting VO_2_max was performed. Race conditions were simulated in the laboratory environment. According to a previously arranged scheme, the participant communicated with the treadmill operator, who could change the speed (increase or reduce it), but not the incline, which at all times stayed at 6%. After the test, a capillary blood sample is taken from the finger to determine the blood lactate concentration. As a result, the lowest load—speed (km/h)—at a 6% incline that induced peak oxygen consumption was sought. At this stage, the participant was evaluated to determine whether the cross-country skier achieved the previously set criteria (VO_2max_ for males > 50 mL/kg/min, and females > 40 mL/kg/min).

To measure physiological and performance changes after two weeks of dynamic high-intensity interval training, the same test protocol was used.

#### 2.2.3. Interval Training

**Figure 2:**
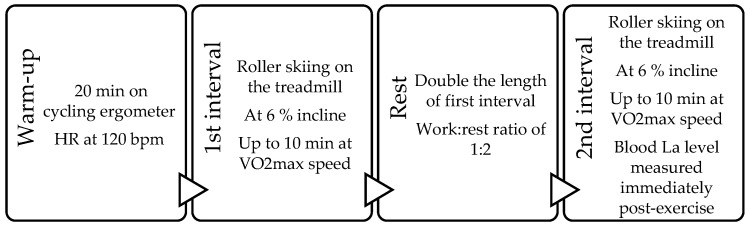
The model for interval training session used in the study.

A dynamic high-intensity interval training protocol of “two times up to 10 min” was used. A total of six training sessions were performed during the two weeks of training. Intervals were performed at the lowest load intensity that reached peak oxygen consumption as determined during initial testing. The participant was instructed to follow subjective feelings and to perform at the intended load until exhaustion. The time spent during the first interval load is registered. In the following rest period before the next (second) interval, the time spent in the first interval is doubled, thus determining the rest period, obtaining a load:rest ratio of 1:2. During the rest period, the participant could choose their actions freely; they were allowed to sit, walk, have a drink, or perform other actions that the participant considered necessary. Next, the participant was instructed to perform a second interval at the same load intensity until exhaustion. After the second interval, the day’s work was completed, and the participant was invited to come to the next training session. See the model of a training session in Figure 2. Each interval training session is followed by at least one day off.

### 2.3. Statistical Analysis

“R stats” R studio (Version 2021.09.2 Build 382, Auckland, New Zealand) computer software was used for data and statistical analysis. Data are presented as mean ± standard deviation (SD). Normality was assessed for each variable using the Shapiro–Wilk test (α = 0.05). If the normality assumption and homogeneity of variances (F-test) were satisfied, parametric tests were used; otherwise, non-parametric equivalents were applied.

For repeated measures across sessions, the Friedman test was used when data violated parametric assumptions; otherwise, repeated-measures ANOVA was considered. Post hoc comparisons between individual sessions were conducted using Wilcoxon signed-rank tests with Bonferroni correction (α = 0.05) when data were non-normal.

Effect sizes for parametric tests were expressed as Cohen’s d, whereas rank-biserial correlation (r_aβ_) was used for non-parametric tests. Correlations between baseline performance and training response were analyzed using Pearson’s r when both variables were normally distributed and Spearman’s ρ otherwise. Stability of VO_2_max post-intervention was evaluated using the two one-sided tests (TOST) equivalence procedure. Finally, a post hoc power analysis for the observed Pearson correlation was conducted using GPower (version 3.1.9.7).

## 3. Results

### 3.1. Performance at VO_2max_

**Figure 3:**
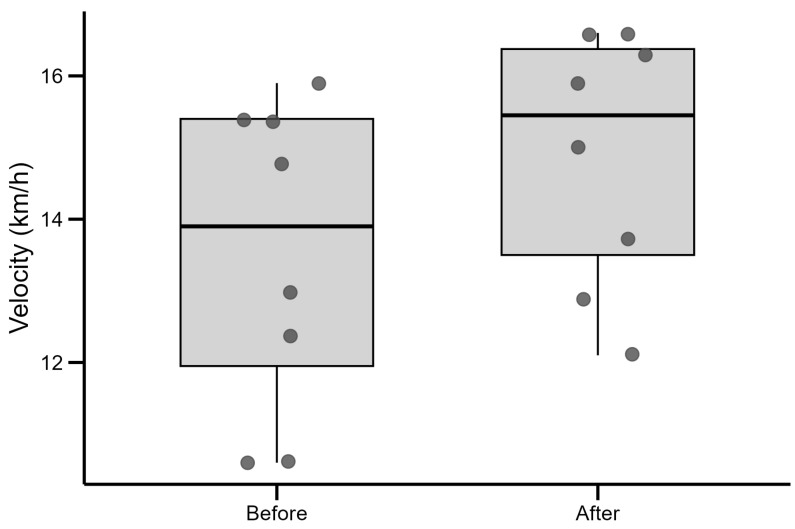
The mean roller skiing speed of the participants on the treadmill before and after two weeks of interval training.

In pre- and post-exercise tests, roller skiing speed at VO_2max_ in km/h was measured. In the pre-test, the speed reached by participants was 13.5 ± 2.16 km/h; in the post-test, it was 14.8 ± 1.7 km/h. The mean increase in speed was 1.4 ± 1.29 km/h after two weeks of training (*p* = 0.0196), as displayed in Figure 3, where each dot represents an individual value. The boxplot shows the interquartile range (IQR): lower edge (Q1): 25th percentile; upper edge (Q3): 75th percentile; middle line: median (50th percentile). The largest gain for one subject was 4.4 km/h: from 10.6 to 15 km/h; re-running the paired *t*-test without this value yielded *p* = 0.0014, confirming that the reported effect is robust to the exclusion of this extreme value. The speed of some participants increased by 0.5 km/h. No one in the post-test reached a lower speed than in the pre-test. The effect size was large with Cohen’s d = 1.06 and Hedge’s g = 0.93. The gain scores violated normality (Shapiro–Wilk *p* = 0.0019). A Wilcoxon signed-rank test confirmed a significant increase in maximum speed (*p* = 0.0078). Gain scores were negatively correlated with baseline speed (Pearson’s *r* = −0.75, *p* = 0.035; Spearman ρ = −0.71, *p* = 0.049), indicating that participants with lower initial speeds experienced grater improvements. Post hoc power analysis showed power of 0.85, which exceeds the conventional threshold of 0.80.

There were no statistically significant changes observed in VO_2max_ itself (*p* > 0.05). the pre-test showed VO_2max_ of 55.3 ± 5.2 mL/kg/min with post-test levels reaching 57.2 ± 5.5 mL/kg/min. The largest gain was 6.9 mL/kg/min: from 47.9 to 54.8 mL/kg/min. Some participants reached lower VO_2max_ levels in the post-test. The largest loss was 3.4 mL/kg/min: from 53.6 to 50.2 mL/kg/min.

A TOST equivalence test (Δ = ±2.8 mL/kg/min, 5% of baseline) confirmed that VO_2max_ did not meaningfully change after the dynamic HIIT (TOST *p* = 0.03).

### 3.2. Metabolic Adaptations

**Figure 4:**
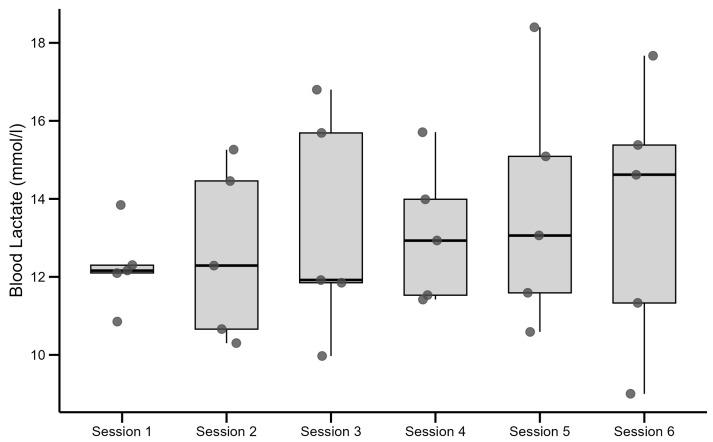
Lactate concentration in the capillary blood immediately post-exercise in six training sessions.

Lactate concentration in the capillary blood was measured after every interval during the training session and stress test. The lactate concentration reached 11.88 ± 1.17 mmol/L in the pre-test and remained 12.03 ± 1.8 mmol/L in the post-test. Formal two one-sided tests (TOST) with a margin of ±2 mmol/L (like SD) for equivalence were performed and resulted in a one-sided *p*-value (0.015). We could conclude equivalence of lactate concentration levels within ±2 mmol/L. All lactate values were significantly above the commonly accepted lactic threshold 2 (LT2) of 4 mmol/L (*p* < 10^−43^) and even exceeded the more stringent 8 mmol/L benchmark (*p* < 10^−26^), shown in Figure 4, where each dot represents an individual value. The boxplot shows the interquartile range (IQR): lower edge (Q1): 25th percentile; upper edge (Q3): 75th percentile; middle line: median (50th percentile).

**Figure 5:**
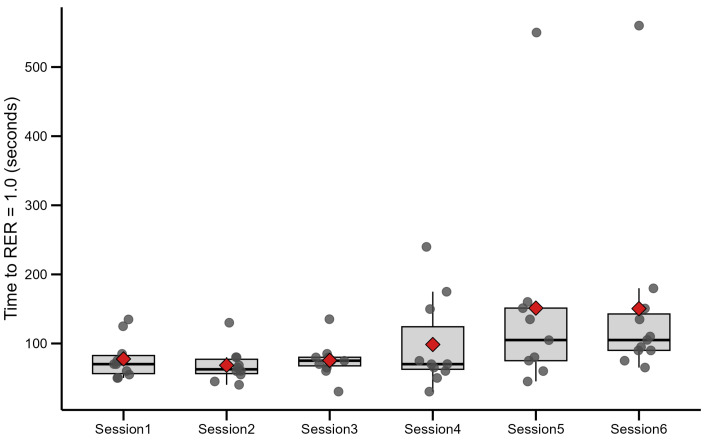
The time to reach a respiratory exchange ratio (RER) value of 1.0 in every interval training session.

Respiratory gas exchange analysis was performed in every interval training session to assess how the participants’ metabolism adapted to use different energy sources. Therefore, the time to reach a respiratory exchange ratio (RER) value of 1.0 was used as a marker. The time to reach RER 1.0 is displayed in Figure 5, where each dot represents an individual value. The boxplot shows the interquartile range (IQR): lower edge (Q1): 25th percentile; upper edge (Q3): 75th percentile; middle line: median (50th percentile). And the diamond represents the average value.

Mean ± SD times to RER 1.0 were 77.5 ± 29.9 s in interval training session No.1 and 150.5 ± 149.2 s in interval training session No.6. The Friedman test showed a significant effect of session on time to RER = 1.0 (χ^2^ (5) = 13.21, *p* = 0.021). Post hoc Wilcoxon signed-rank testing with Bonferroni correction confirmed a significant increase from training session No.1 to session No.6 (S = 5.0, *p* = 0.0195; *p* = 0.020).

### 3.3. Time to Exhaustion

**Figure 6:**
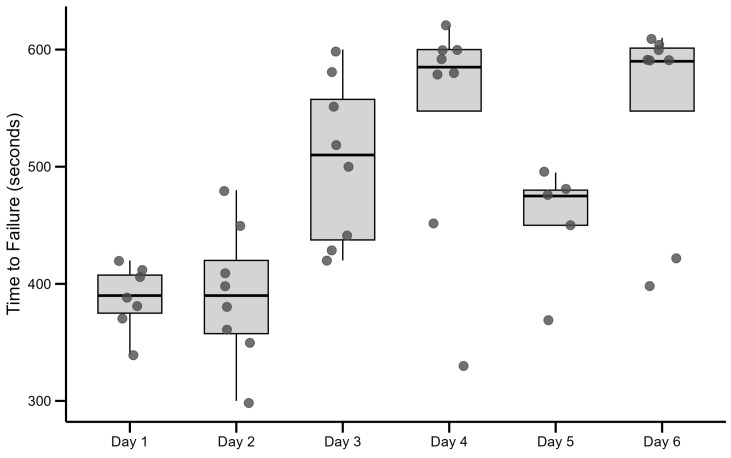
The time to exhaustion in every interval training day on treadmill, first session.

As the dynamic high-interval training protocol requires the subject to execute each interval to the point of failure or exhaustion, it is highly informative to measure the time to failure from session to session, as this represents possible adaptations to high-intensity exercise tolerance. In our study, the mean time passed to exhaustion at the first interval session was 357 ± 53 s. By the sixth session, this time has increased by 132 s to 535 ± 87 s or 32.9%, as shown in Figure 6, where each dot represents individual value. The boxplot shows the interquartile range (IQR): lower edge (Q1): 25th percentile; upper edge (Q3): 75th percentile; middle line: median (50th percentile).

### 3.4. Changes in Body Mass

Before the HIIT, participants’ mean body mass was 70.975 ± 8.200 kg, but after the HIIT, it was 69.912 ± 8.000 kg; the mean body mass reduction was 0.538 ± 0.480 kg (*p* = 0.016).

The largest body mass loss was 1.3 kg: from 62.6 to 61.3 kg in the two weeks. A Cohen’s *d* of ∼1.1 indicates a large effect, and Hedges’ *g* (which adjusts for small sample size) confirms this (∼0.97).

## 4. Discussion

The primary finding of this study is that a novel two-week dynamic HIIT protocol (“two times up to ten minutes”) significantly improved maximal roller skiing speed in trained cross-country skiers, even in the absence of a statistically significant increase in VO_2_max. This suggests that performance improvements can occur independently of changes in maximal oxygen uptake, due to enhanced metabolic efficiency, neuromuscular coordination, and psychological adaptations.

### 4.1. Performance and Efficiency

The significant increase in roller skiing speed at VO_2max_ (*p* = 0.0196; Cohen’s d = 1.06) with a robust effect even after exclusion of an outlier suggests a strong adaptation to this training model. Notably, individuals with lower baseline speed experienced the greatest performance gains, highlighting the potential of this protocol to be particularly effective in athletes with more room for improvement.

All participants had limited prior experience training on treadmills, and although a familiarization session was provided, it may have been insufficient for full adaptation to the testing environment. The athlete with the largest improvement (+4.4 km/h) likely had the steepest learning curve, with the initial test underestimating true performance capacity due to lack of treadmill familiarity. As stated above, excluding this data point did not alter the statistical significance of the main findings, indicating that the observed training effect was robust. Nonetheless, these results highlight the importance of adequate familiarization when testing trained athletes using equipment outside their usual training setting.

McLaughlin et al. [29] revealed in their study that more than 80% of 16 km race runners’ endurance performance variance in exercises was related to the athletes’ VO_2max_; Coyle [30] found that this could not be observed in well-trained endurance athletes because the increase in VO_2max_ was very small, and often no relationship between VO_2max_ and performance was observed. Athletes’ performance in endurance events with a duration of more than a few minutes can be improved by training without changes in VO_2max_ but due to an increase in the mechanical work economy [31,32]. Elite athletes trained in endurance sports, such as marathon runners, have a high VO_2max_ in the early years of their sports career and later improve their long-term performance due to better mechanical work economy [33]. Ma et al. [34] determined in their review article that HIIT programs were more effective than conventional training methods in improving aerobic power (VO_2max_) and promoting aerobic performance in elite athletes. This could be explained by different interpretations of the elite athletes’ definition (an athlete must be involved in regular competition at the national level); they do not necessarily mean they were top-level athletes.

The majority of energy during exercise lasting longer than five minutes was provided by the aerobic system, and lactate clearance improvement also played a significant role. Therefore, many studies have confirmed that intensified training loads enhance trial time in a 10 km race, which was associated with a higher anaerobic capacity without changes in VO_2max_ [31,35]. Llanos-Lagos et al. [36] proved that even strength training (i.e., high-load training, submaximal load training, and/or plyometric training) with high loads can improve performance (i.e., time trial, time to exhaustion) in middle-distance and long-distance runners without significant changes in VO_2max_.

Although VO_2max_ itself did not increase significantly, equivalence testing confirmed that changes remained within a narrow margin (±2.8 mL/kg/min), indicating that the protocol had no meaningful effect on peak oxygen uptake. This reinforces the notion that performance gains can be mediated through other mechanisms such as improved movement economy or lactate tolerance, as has been shown in both runners [6] and cyclists [5].

### 4.2. Metabolic Adaptations

The session-by-session analysis—specifically tracking changes in time to RER = 1.0, time to failure, and post-exercise blood lactate concentration—enabled us to monitor within-cycle adaptations in both performance and metabolic efficiency. This approach aligns with recent proposals to move beyond isolated pre–post assessments and instead model dynamic internal–external load relationships across training microcycles. Recent publications have emphasized the importance of such frameworks, integrating physiological and biomechanical responses to better understand adaptation and fatigue in endurance athletes [37]. While their model includes biomechanical fatigue indicators such as gait variability, these are not directly applicable to skiing. Nonetheless, our use of repeated internal load markers—including metabolic (RER, lactate) and performance (time to failure) variables—offers a sport-specific representation of training efficiency and adaptation over the course of the HIIT shock cycle.

The significant increase in time to reach RER = 1.0 (*p* = 0.021) was a finding of particular importance, indicating a shift toward greater reliance on aerobic metabolism before crossing VT2. It can be interpreted as indicative of enhanced fat oxidation capacity and delayed carbohydrate reliance—key features of aerobic metabolic adaptation. These findings are in line with other research that improved fat oxidation [38].

This research shows increased metabolic efficiency and improved substrate utilization in aerobic reactions, both key targets of the HIIT method, and shows similar findings to mitochondrial function research by Granata and colleagues [39]. Despite consistently high lactate values, equivalence testing confirmed stability of this parameter, suggesting that participants maintained a high-intensity output throughout the intervention. A similar RER decrease due to two different HIIT protocols was observed in endurance athletes, but not in sprint-trained athletes, in the investigation of Cipryan et al. [40].

### 4.3. Body Mass and Training Load

A modest but significant reduction in mean body mass (−0.54 kg, *p* = 0.016) was observed, which may be relevant for athletes seeking to optimize power-to-weight ratios; nevertheless, it was not a primary focus of this study. The large effect size suggests the potential usefulness of this protocol to reduce athletes’ body mass through increased energy expenditure and post-exercise oxygen consumption. Our results tend to agree with the meta-analysis data by Khodadadi et al., which demonstrated favorable body composition outcomes following all modalities of HIIT programs (cycling, overground running, treadmill running), with overall reductions in body fat in percentage (%) and body fat mass and improved body fat-free mass in the participants [41]. Body composition was not measured in this study, so the findings are of little value.

### 4.4. Time to Exhaustion

The progressive increase in time to exhaustion across the six training sessions—from 357 ± 53 s in session No.1 to 535 ± 87 s in session No.6 (≈32.9% relative improvement)—provides strong evidence of adaptation to the dynamic HIIT protocol. This enhanced ability to sustain a fixed supramaximal load likely reflects improvements in high-intensity exercise tolerance, fatigue resistance, and more efficient lactate clearance.

Notably, VO_2_max remained stable (within the equivalence bounds), suggesting that the gains in exhaustion time were not driven by central aerobic capacity but rather by peripheral adaptations. These include increased mitochondrial content, elevated oxidative enzyme activity, enhanced capillarity, and better metabolic by-product clearance. Such changes are supported by recent HIIT studies. For example, Li et al. found that, compared to moderate continuous training, HIIT produced greater mitochondrial adaptations, even when VO_2_max increases were modest [42]. Hung et al. similarly emphasize HIIT’s role in boosting fatigue resistance through repeated high-intensity efforts and metabolic enzyme improvements [43]. The meta-analysis by Mølmen et al. showed that high-intensity interval training (HIIT) and sprint interval training significantly enhance mitochondrial content and capillarization in skeletal muscle [44].

Furthermore, the individualized format of our dynamic protocol—where athletes self-terminate intervals by reaching exhaustion rather than working fixed intervals—may have facilitated maximal engagement of fatigue-sensitive systems each session. This likely contributed to the strong gains in exhaustion time without needing concurrent increases in VO_2_max. These findings align with prior work showing that time to exhaustion can increase independently of VO_2_max when training targets neuromuscular, metabolic, and buffering adaptations [45].

### 4.5. Limitations of the Study

This study has two main limitations. First, the lack of a control group limits causal inference. This limitation has been observed in many different studies, especially recent studies involving gene expression [46]. However, the single-group, paired-sample design is commonly used in short-term training interventions and allows for sensitive detection of within-subject changes while controlling for inter-individual variability [47,48]. The short intervention period and standardized conditions further help to minimize the influence of external variables. Second, the relatively small sample size reduces statistical power and may limit generalizability, particularly to broader athletic populations. Nonetheless, small samples are typical in HIIT studies involving tightly controlled protocols or trained individuals, and several prior investigations have reported meaningful physiological adaptations using similar or smaller cohorts [21,23]. These findings should therefore be viewed as exploratory and hypothesis-generating, warranting confirmation in larger, controlled trials.

The research hypothesis was partially confirmed: the implementation of a novel dynamic HIIT protocol, combining fixed-load and variable-time intervals, resulted in a higher speed of skiing, metabolic adaptation to aerobic pathways, and an increase in the time to exhaustion. Nevertheless, VO_2max_ did not change. It improved the aerobic performance characteristics in qualified cross-country skiers.

A further limitation is that we did not monitor caloric intake, macronutrient composition, or changes in body composition throughout the two-week HIIT intervention, including the rest period between intervals. Fluid intake was not standardized and carbohydrate consumption during recovery cannot be excluded as a potential confounding factor. Although participants were instructed to maintain habitual diets, any unintentional energy deficits—common with increased training load—could have occurred. Such deficits are known to contribute to rapid weight loss and may independently enhance fat oxidation and metabolic efficiency [49]. Evidence shows that high-intensity exercise often leads to spontaneous reductions in energy intake or shifts in food choices [50], and there is research that shows HIIT can help reduce muscle loss in calorically restricted individuals [51]. Without food logs, energy expenditure measures, or body weight tracking, we cannot exclude the potential confounding effects of nutritional intake on our primary outcomes. Future studies should prioritize structured dietary monitoring—such as standardized meals or validated food diaries—to disentangle the specific effects of training from nutritional influences.

Also, we did not directly monitor or quantify each athlete’s external training load outside of these prescribed intervals. All participants continued their regular endurance and technical workouts as directed by their coaches, but variations in volume, intensity, and recovery practices could have influenced the observed adaptations. Future studies should incorporate objective monitoring (e.g., training logs, wearable load-tracking devices) of all concurrent training to more precisely isolate the effects of the HIIT block [52].

### 4.6. Practical Implementation

The two-week, six-session HIIT shock cycle evaluated in this study exemplifies a potent yet brief overload microcycle, which can be strategically deployed within larger periodized training frameworks. Evidence suggests that, following such an overload, a taper—defined as a 7–14-day reduction in training volume by approximately 40–60%, while preserving intensity and frequency—can yield meaningful performance gains (~2–8%) in endurance athletes [53,54]. For example, a recent meta-analysis found that tapering combined with pre-taper overload significantly enhanced time-trial and time-to-exhaustion performance compared to taper alone [53]. Additionally, focused shock-cycle HIIT has been shown to induce rapid mitochondrial and aerobic adaptations when followed by adequate recovery periods [55]. Practically, implementing our six-session HIIT block during the final base phase, then reducing volume but maintaining intensity in the subsequent two-week taper, offers a time-efficient and evidence-based approach for pre-competition preparation or mid-season reloading.

## 5. Conclusions

This study demonstrates that a short-term, dynamic high-intensity interval training (HIIT) protocol—characterized by fixed-load, variable-time intervals—can elicit significant improvements in maximal roller skiing speed and metabolic efficiency in well-trained cross-country skiers and an increase in the time to exhaustion. These characteristics of performance gains occurred without significant VO_2max_ changes, so highlighting the importance of peripheral and neuromuscular adaptations beyond central cardiovascular improvements.

The observed enhancements (prolongation) in time to reach the respiratory exchange ratio (RER) value of 1.0, together with the significant reductions in the athletes’ body mass, further support the effectiveness of this protocol in improving metabolic flexibility and overall conditioning. Importantly, the training model’s individualization—allowing participants to perform the training interval load to exhaustion and recover proportionally—may contribute to its adaptability and effectiveness across varying performance levels.

These findings support the inclusion of dynamic HIIT as a complementary tool in endurance training programs, particularly in periods of performance plateaus or when time-efficient interventions are needed. Future studies are necessary to explore long-term outcomes, compare dynamic versus traditional HIIT protocols, with control groups, external load monitoring, and focus on body composition and nutritional intake, and evaluate their application in sport-specific environments.

## Data Availability

Data supporting reported results can be found at https://doi.org/10.5281/zenodo.15733931 (accessed on 10 October 2025).

## Abbreviations

The following abbreviations are used in this manuscript:

VO_2max_	Maximum oxygen uptake
HIIT	High-intensity interval training
RER	Respiratory exchange ratio
TOST	Two one-sided tests
EPOC	Excess post-exercise oxygen consumption
CP	Critical power
VT2	Ventilatory threshold 2
LT2	Lactic threshold 2
